# Human papillomavirus infection in Shanxi Province, People's Republic of China: a population-based study

**DOI:** 10.1038/sj.bjc.6603208

**Published:** 2006-06-13

**Authors:** M Dai, Y P Bao, N Li, G M Clifford, S Vaccarella, P J F Snijders, R D Huang, L X Sun, C J L M Meijer, Y L Qiao, S Franceschi

**Affiliations:** 1 International Agency for Research on Cancer, 150 cours Albert Thomas, 69372 Lyon cedex 08, France; 2 Cancer Institute/Hospital, Chinese Academy of Medical Sciences, 17, South Pan Jia Yuan LN, PO Box 2258, Beijing 100021, China; 3 Vrije University Medical Center, Postbus 7057, 1007 MB, Amsterdam, The Netherlands; 4 Yangcheng Tumor Hospital, 4 Qiaodong Road, Yangcheng 048100, Shanxi, China; 5 Department of Gynecological Oncology, Shanxi Provincial Tumor Hospital, 3 Zhigongxincun, Taiyuan 030013, Shanxi, China

**Keywords:** human papillomavirus, cervical neoplasia, China, epidemiology

## Abstract

To investigate the prevalence of, and risk factors for, cervical infection with human papillomavirus (HPV) in the rural province of Shanxi, People's Republic of China, which has relatively high cervical cancer mortality rates, we interviewed and obtained cervical cell samples from 662 women aged 15–59 years. A total of 24 different HPV types were identified using a GP5+/6+-based PCR assay able to detect 44 different HPV types. Human papillomavirus prevalence was 14.8% overall and 9.6% among women without cervical abnormalities (14.2 and 8.9%, respectively, age standardised to the world standard population). Multiple-type infections accounted for 30.6% of all infections. By far the most commonly found type was HPV16 (5.7% of all women and 38.8% of HPV-positive women), followed by HPV 58, 52, 33 and 18. Unlike most previous studies published, HPV prevalence was lower among women younger than 35 years (8.7%) than those older than 35 years (17.8%). High-risk HPV types predominated in all age groups. Although low-risk HPV types were rare in young women, they became more common with increasing age. 92.3% of women with cervical intraepithelial neoplasia grade 3 were infected with high-risk HPV types, but none with low-risk types only. No significant difference in HPV positivity was observed by educational level, sexual habits, reproductive history or use of contraceptive methods in this rural low-income Chinese population.

Following acceptance of human papillomavirus (HPV) as a necessary cause of cervical cancer, primary and secondary cervical cancer prevention strategies have moved towards detection and control of the virus. In order to understand the potential impact of HPV vaccination and HPV-based screening, epidemiological data on HPV type-specific prevalence among women from different populations is important. The International Agency for Research on Cancer (IARC) has therefore carried out surveys in representative samples of women worldwide (Jacobs *et al*, 2000; Lazcano-Ponce *et al*, 2001; Molano *et al*, 2002; Anh *et al*, 2003; de Sanjosé *et al*, 2003; Matos *et al*, 2003; Shin *et al*, 2003; Sukvirach *et al*, 2003; Ferreccio *et al*, 2004; Shin *et al*, 2004; Thomas *et al*, 2004; Franceschi *et al*, 2005; Ronco *et al*, 2005). These surveys have disclosed wide variations in HPV prevalence and some in the relative frequency of individual HPV types (Clifford *et al*, 2005).

The population of the People's Republic of China is 1.3 billion, of which 0.9 billion is rural. According to data from a network of 10 Chinese cancer registries, cervical cancer incidence in China is estimated to be below 4/100 000 (Parkin *et al*, 2002). However, nationwide mortality surveys show a heterogeneous pattern of cervical cancer risk, with particularly high rates in central rural provinces (Yang *et al*, 2003a). Furthermore, while cervical cancer mortality appears to have declined considerably in urban China between 1987 and 1999, this is less marked in rural areas (Yang *et al*, 2003b). A recent study in one such high-risk rural area, Shanxi Province, revealed that 23.6% of women aged 35–50 years were infected with high-risk HPV types (Zhao *et al*, 2006). However, at present no population-based data are available on HPV prevalence across a broader age range of Chinese women, nor on the relative frequency of individual HPV types.

## MATERIALS AND METHODS

### Study subjects

This study was a collaborative project between the IARC and the Cancer Institute of the Chinese Academy of Medical Sciences (CI-CAMS) and study methods were similar to those used for previous IARC HPV prevalence surveys (Clifford *et al*, 2005). The study area of Shanxi Province is a rural, mountainous area in central China, with a population mainly employed in agriculture and mining.

A total of 2229 women aged 15–59 years were enumerated in March and April 2004 from the population list of eight randomly chosen villages in Hebei Commune, Yangcheng County, Shanxi Province, China. The study purpose was to enroll approximately 100 women in each 5-year age group between 15–19 and 55–59 years. Women were contacted at their home by village doctors and invited to Yangcheng Tumour Hospital to participate in the study. All mentally and physically competent women aged 15–59 years were eligible for the study. Women were interviewed regardless of their marital status but pelvic examination and cervical cell specimen collection were only possible among married women.

Of the 2229 enumerated women, 157 were not found at the address given on the population list, and 404 were not invited as the required sample size for their age group had been reached. Of the 1668 invited women, 727 (43.6%) did not participate in the study, mainly because they said they did not have time or did not understand the need to undergo gynaecological examination in the absence of symptoms. Of the remaining 941 women who participated between May and June 2004, 197 did not give a cervical cell specimen (159 unmarried, 13 pregnant, nine menstruating, and three hysterectomised women, as well as 13 married women who opted not to undergo pelvic examination).

The interview was administered by one of four research nurses in the Shanxi dialect of Chinese. The structured questionnaires included information on socio-demographic characteristics, reproductive and menstrual factors, sexual habits of women and their husbands, and lifetime use of contraceptive methods.

All participants signed an informed consent form according to the recommendations of the IARC and CI-CAMS ethical review committees, which approved the study.

### Gynaecological examination and cervical specimen collection

A total of 744 married women underwent a pelvic examination by one of four gynaecologists. Firstly, a sample of exfoliated cervical cells for liquid-based cytology and HPV testing was collected. A cytobrush was inserted into the endocervical canal and rotated gently 180^o^ in order to collect cells from the endo- and ectocervix. The brush containing cellular material was then placed in a vial containing CytoRich transport media (Tripath Imaging, Burlington, NC, USA). Two aliquots of cellular material were obtained in the CI-CAMS laboratory using a strict protocol to minimise contamination, and were then sent to IARC for HPV testing. Remaining cellular material was used to perform liquid-based cytology at CI-CAMS. Secondly, the cervix was inspected by visual inspection with acetic acid (VIA) and visual inspection with Lugol's Iodine solution (VILI), followed by a digital colposcopy. Women with suspected abnormalities at VIA, VILI or colposcopy had a colposcopy-directed biopsy taken, and women whose entire squamocolumnar junction could not be visualised underwent endocervical curettage. When liquid-based cytology results became available, all women with cytologically abnormal lesions (atypical squamous cells of undetermined significance (ASCUS) or worse) not detected at VIA, VILI or digital colposcopy, were recalled and had biopsies taken. Treatment of detected lesions was performed according to local protocols using loop electrosurgical excision procedures.

### Cytology and histology

Liquid-based cytology was performed at CI-CAMS. Slides were prepared from the CytoRich preserved specimen according to the manufacturer's standard protocol (Belinson *et al*, 2003), and results classified according to the Bethesda System. At CI-CAMS all abnormal smears, as well as 10% of normal smears chosen at random, were reviewed by an experienced cytologist, and cervical biopsies by a pathologist.

On account of the performance of multiple screening tests and the especially high proportion of histological confirmation of abnormal cervical findings at VIA, VILI, digital colposcopy or liquid-based cytology, cervical abnormalities were defined in the present study as presence of histologically confirmed cervical intraepithelial neoplasia (CIN)1 or worse.

### HPV detection

Human papillomavirus testing was performed on exfoliated cervical cells in the Department of Pathology at the Vrije University Medical Center, Amsterdam, The Netherlands. To that end DNA was extracted first from the remainder of the CytoRich sample using a High Pure PCR product Purification Kit according to the manufacturer's recommendations (Roche Applied Science, Mannheim, Germany). *β*-Globin PCR analysis was performed firstly to confirm the presence of human DNA in all specimens (Roda Husman *et al*, 1995). The overall presence of HPV DNA was determined by performing a general primer GP5+/6+-mediated PCR, which permits the detection of a broad spectrum of genital HPV types at the subpicogram level (Jacobs *et al*, 2000). Human papillomavirus positivity was assessed by hybridisation of PCR products in an enzyme immunoassay using two HPV oligoprobe cocktails that, together, detect the following 44 HPV types: HPV 6, 11, 16, 18, 26, 30, 31, 32, 33, 34, 35, 39, 40, 42, 43, 44, 45, 51, 52, 53, 54, 55, 56, 57, 58, 59, 61, 64, 66, 67, 68, 69, 70, 71 (equivalent to CP8061), 72, 73, 81 (equivalent to CP8304), 82 (IS39 and MM4 subtypes), 83 (equivalent to MM7), 84 (equivalent to MM8), cand85, 86, cand89 (equivalent to CP6108) and JC9710. Subsequent HPV typing was performed by reverse line blot hybridisation of PCR products, as described previously (van den Brule *et al*, 2002).

Human papillomavirus types considered high-risk for this analysis included HPV 16, 18, 31, 33, 35, 39, 45, 51, 52, 56, 58, 59, 68, 73 and 82 (Muñoz *et al*, 2003). All other HPV types were considered low risk.

### Statistical analysis

Odds ratios (ORs) for HPV positivity and corresponding 95% confidence intervals (CIs) were calculated by means of unconditional logistic regression equations, adjusted for age (15–24, 25–34, 35–44, 45–54, 55–59 years). The statistical significance of trends for ORs was assessed by considering the categorical variables as a continuous variable in the logistic model.

## RESULTS

Of 744 married women who provided cervical cell samples, nine had inadequate cytology results and 73 had *β*-globin-negative samples, leaving 662 women with valid cytology and HPV results. Among them, 68 (10.3%) had histologically confirmed cervical abnormalities, including 39 CIN1, 16 CIN2, 12 CIN3 and one squamous cell carcinoma.

The prevalence of any HPV type was 14.8% (60.3% and 9.6% among women with and without cervical abnormalities, respectively, Table 1). The corresponding prevalences age-standardised to the world population were 14.2% overall, and 8.9% among those without cervical abnormalities. In total, 68 women had single-type and 30 had multiple-type infections. In all, 24 individual HPV types were identified.

High-risk HPV types were substantially more frequent (12.2% of all women) than low-risk types (3.8%). The most common types in either single- or multiple-type infections were HPV16 (5.7%), 58 (3.2%), 52 (1.2%), 33 (1.2%), 42 (a low-risk type, 1.1%), but HPV type distribution varied by the presence of cervical abnormalities. High-risk HPV types were found in 54.4% of women with cervical abnormalities and in 11 out of 12 (91.7%) women with CIN3. The only woman identified with squamous cell carcinoma was HPV16 positive.

**Figure 1:**
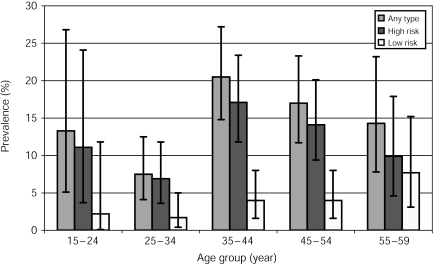
Age-specific prevalence of cervical HPV DNA and corresponding 95% CI. Shanxi Province, China, 2004.

Figure 1 and Table 2 show the prevalence of HPV (any type, high- and low-risk types, separately) by age group. No significant trend in prevalence by age group emerged for any HPV type, nor for high-risk HPV types. Low-risk HPV types were found very rarely among women younger than 35 years, but their frequency increased significantly with age (*P* for trend=0.03). For both high-risk and low-risk types, lowest prevalence was found in the 25–34 year age group. Table 2 also shows the association between the presence of cervical abnormalities and different HPV types. Positivity for high-risk HPV types was strongly associated with increasing cervical lesion severity (OR for CIN3 *vs* normal=136; 95% CI: 17.2–1082). Positivity for low-risk HPV types was associated with CIN1 and CIN2, but not CIN3 (Table 2).

Table 3 shows the relationship between HPV positivity and various characteristics of study women after adjustment for age. No significant association was found between HPV positivity and education level, age at first sexual intercourse, lifetime number of sexual partners, husband's extramarital sexual relationships, age at menarche or menopause, number of births, or the use of the two most widespread contraceptive methods in the study area (i.e. intrauterine device and tubal ligation). Only 17.7% of study women reported two or more sexual partners, and 24.2% reported sexual debut before age 19 years. No significant associations arose when we evaluated correlates of high-risk HPV positivity only (data not shown).

Other characteristics that were examined and found not to be associated with HPV positivity included multiple marriages (50 women, OR=0.4; 95% CI: 0.2–1.3), being a widow (24 women, OR=1.1; 95% CI: 0.3–3.2), history of spontaneous abortion (98 women, OR=0.9; 95% CI: 0.5–1.7), and induced abortion (241 women, OR=0.8; 95% CI: 0.5–1.2). No study woman reported divorce or cigarette smoking. Use of oral contraceptives and condom was rare (21 and 14 women, respectively) and was associated with ORs of 2.0 (95% CI: 0.7–5.4) and 0.5 (95% CI: 0.1–3.7), respectively. Inflammation and/or infection upon colposcopy was found in 55% of women, but was not significantly associated with HPV infection (OR 1.3, 95% CI: 0.8–2.0). In all, 64 women reported to have had at least one previous cytological smear (OR for HPV positivity=0.5; 95% CI: 0.2–1.2, data not shown).

## DISCUSSION

Relative to previous population-based surveys of HPV worldwide, noteworthy findings from Shanxi Province, China, include a high prevalence of HPV infection, a marked predominance of HPV16 among HPV-positive women, and the lack of an observable decline in HPV prevalence with increasing age.

Age-standardised HPV prevalence in Shanxi Province was found to be similar to that found in other high-risk areas for cervical cancer studied by IARC using the same HPV testing protocol in Latin America (Molano *et al*, 2002; Matos *et al*, 2003; Ferreccio *et al*, 2004) and India (Franceschi *et al*, 2005), although lower than in some areas of sub-Saharan Africa (Thomas *et al*, 2004). In contrast to previous studies in high-resource countries (Peto *et al*, 2004), however, no decline of HPV prevalence was seen with increasing age, and highest HPV prevalence was found among middle-aged women. This age pattern is similar to the flat age curve seen in rural India (Franceschi *et al*, 2005) and Africa (Thomas *et al*, 2004; Clifford & Franceschi, 2005).

Large cohort studies in Colombia (Muñoz *et al*, 2004) and Costa Rica (Castle *et al*, 2005) have shown that new HPV infection in middle-aged women is not rare, but, Castle *et al* (2005) suggested a stronger role for HPV persistence than acquisition of new infection in determining HPV prevalence in women over 45 years of age. Thus, high HPV prevalence in middle-aged women in Shanxi Province may indicate a relative lack of clearance or high frequency of reactivation of HPV infection. Furthermore, in conservative societies like rural China, it is likely that young women are not more frequently exposed to HPV than middle-aged women. Indeed, we observed no differences in indicators of sexual behaviour (e.g. age at first sexual intercourse (19.4 and 19.0 years, respectively) or frequency of husband's extramarital sexual relationships (15.2 and 14.0%, respectively)) among women aged under 25 years and over 45 years.

Our survey is the first population-based study in China to assess HPV type-specific prevalence across a broad age-range of women. It showed that HPV16 is the most frequently found type in Shanxi Province, affecting 38.8% of HPV-positive women. The corresponding percentages in other IARC HPV prevalence surveys in different continents were 32.6% in Europe, 22.5% in Asia, 22.0% in Latin America, and 12.2% in Africa (Clifford *et al*, 2005), making the proportion of HPV16-positive women in our present population-based study among the highest recorded worldwide. In a study of 809 cervical cancer cases from four areas in China, a relatively high proportion (66.9%) were positive for HPV16 (Lo *et al*, 2002).

HPV58 was the second most common HPV type in Shanxi Province, and was found in one quarter of all HPV-positive cervical abnormalities, confirming the importance of HPV58 infection in Asia (Chan, 2005). Studies of cervical cancer cases from China have reported varying HPV58 prevalence between 2.9 and 27.5% (Clifford *et al*, 2003). The distribution of other commonly identified HPV types in Shanxi Province (e.g. HPV18, 33 and 42), largely mimics those reported in a previous pooled analysis of IARC HPV prevalence surveys (Clifford *et al*, 2005).

No questionnaire-based information, including sexual habit indicators, reproductive and menstrual characteristics, oral contraceptive or condom use, showed any significant associations with HPV positivity. This may be partly related to the fact that this study took place in a rather homogenous, low-income rural population.

Our present study has strengths and limitations. Among the former, the use of highly sensitive PCR assays and enrolment of a representative sample of the married female population. A high proportion of histological confirmation was also an asset, allowing a clear distinction between the similar potential of high-risk and low-risk types to induce CIN1 and 2 but not CIN3. The main limitation of the present study was that only approximately half of the initially invited population had cells collected for HPV testing. Of the nonparticipants that were interviewed, a large proportion was unmarried. Indeed HPV testing was offered only to married women, who were expected to be a reasonably good proxy of sexually active women in this conservative society. Furthermore, as HPV infection is asymptomatic and does not show, contrary to cervical cancer (Rajkumar *et al*, 2003), any strong socio-economic gradient, it is unlikely that participation was related to women's HPV infection status.

In conclusion, our study allowed the disclosure of a high prevalence of HPV and an age-specific prevalence curve that is in sharp contrast with the peak in HPV prevalence below age 25 years observed in Europe (de Sanjosé *et al*, 2003; Peto *et al*, 2004; Ronco *et al*, 2005) and North America (Bauer *et al*, 1993).

## Figures and Tables

**Table 1:** Prevalence of various HPV types by presence of cervical abnormalities and overall among 662 women. Shanxi Province, China, 2004

**Cervical abnormalities**									
	**Absent**			**Present^a^**			**Total**		
**HPV types**	**Single**	**Multiple**	**Total (%)**	**Single**	**Multiple**	**Total (%)**	**Single**	**Multiple**	**Total (%)**
*Negative*	−	−	537 (90.4)	−	−	27 (39.7)	−	−	564 (85.2)
*Positive*									
Any	42	15	57 (9.6)	26	15	41 (60.3)^b^	68	30	98 (14.8)
High risk	31	13	44 (7.4)	23	14	37 (54.4)	54	27	81 (12.2)
Low risk	10	6	16 (2.7)	2	7	9 (13.2)	12	13	25 (3.8)
X	1	0	1 (0.2)	1	0	1 (1.5)	2	0	2 (0.3)
									
*High risk*									
16	10	8	18 (3.0)	14	6	20 (29.4)	24	14	38 (5.7)
58	7	4	11 (1.9)	4	6	10 (14.7)	11	10	21 (3.2)
52	3	2	5 (0.8)	1	2	3 (4.4)	4	4	8 (1.2)
33	1	2	3 (0.5)	3	2	5 (7.4)	4	4	8 (1.2)
18	3	0	3 (0.5)	0	2	2 (2.9)	3	2	5 (0.8)
56	1	3	4 (0.7)	0	1	1 (1.5)	1	4	5 (0.8)
39	1	2	3 (0.5)	0	1	1 (1.5)	1	3	4 (0.6)
51	1	2	3 (0.5)	0	1	1 (1.5)	1	3	4 (0.6)
45	1	1	2 (0.3)	0	2	2 (2.9)	1	3	4 (0.6)
31	3	0	3 (0.5)	0	0	0	3	0	3 (0.5)
59	0	0	0	1	1	2 (2.9)	1	1	2 (0.3)
68	0	0	0	0	1	1 (1.5)	0	1	1 (0.2)
82	0	0	0	0	1	1 (1.5)	0	1	1 (0.2)
									
*Low-risk*									
42	1	0	1 (0.2)	0	6	6 (8.8)	1	6	7 (1.1)
66	3	1	4 (0.7)	1	0	1 (1.5)	4	1	5 (0.8)
6	2	1	3 (0.5)	0	0	0	2	1	3 (0.5)
40	0	3	3 (0.5)	0	0	0	0	3	3 (0.5)
67	2	0	2 (0.3)	0	1	1 (1.5)	2	1	3 (0.5)
43	1	1	2 (0.3)	0	0	0	1	1	2 (0.3)
53	0	1	1 (0.2)	1	0	1 (1.5)	1	1	2 (0.3)
44	1	0	1 (0.2)	0	0	0	1	0	1 (0.2)
54	0	1	1 (0.2)	0	0	0	0	1	1 (0.2)
55	0	0	0	0	1	1 (1.5)	0	1	1 (0.2)
64	0	1	1 (0.2)	0	0	0	0	1	1 (0.2)

a Includes all histologically confirmed cervical intraepithelial neoplasia (CIN)1 and worse;

b Among 12 women with CIN3, six were positive for HPV16, one for HPV58, one each for HPV16 and 45, HPV16 and 68, HPV45 and 52, HPV51 and 59; one woman with squamous cell carcinoma was HPV16 positive.

**Table 2:** Detection of cervical HPV any type, high-risk types and low-risk types according to age and the presence of cervical abnormalities among 662 women. Shanxi Province, China, 2004

		**HPV positive**								
		**Any**			**High risk^a^**			**Low risk^b^**		
	**Total no.**	**No.**	**%**	**OR (95% CI)^c^**	**No.**	**%**	**OR (95% CI)^c^**	**No.**	**%**	**OR (95% CI)^c^**
*Age (years)*										
15–24	45	6	13.3	1.9 (0.7–5.3)	5	11.1	1.7 (0.6–5.0)	1	2.2	1.3 (0.1–12.7)
25–34^d^	173	13	7.5	1	12	6.9	1	3	1.7	1
35–44	176	36	20.5	3.2 (1.6–6.2)	30	17.1	2.8 (1.4–5.6)	7	4.0	2.3 (0.6–9.2)
45–54	177	30	17.0	2.5 (1.3–5.0)	25	14.1	2.2 (1.1–4.5)	7	4.0	2.3 (0.6–9.2)
55–59	91	13	14.3	2.1 (0.9–4.6)	9	9.9	1.5 (0.6–3.6)	7	7.7	4.7 (1.2–18.7)
* p for trend*				=*0.11*			=*0.34*			=*0.03*
										
*Presence of cervical abnormalities*										
Normal^d^	594	57	9.6	1	44	7.4	1	16	2.7	1
CIN1	39	18	46.2	7.6 (3.7–15.4)	16	41.0	8.6 (4.1–18.0)	6	15.4	5.5 (2.0–15.5)
CIN2	16	11	68.8	22.0 (7.2–67.4)	9	56.3	16.8 (5.8–48.4)	3	18.8	8.8 (2.2–35.1)
CIN3 and worse	13	12	92.3	102 (12.8–803)	12	92.3	136 (17.2–1082)	0	0	0 (0–11.4)
*p for trend*				<*0.001*			<*0.001*			=*0.007*

a Includes that in multiple infections with low-risk types.

b Includes that in multiple infections with high-risk types.

c Adjusted for age (10-year groups);

d Reference category. OR=odds ratio; CI=confidence interval; CIN=cervical intraepithelial neoplasia.

**Table 3:** Detection of cervical HPV DNA according to selected characteristics among 662 women. Shanxi, China, 2004

		**HPV DNA positive**		
**Characteristics**	**No. of women**	**No.**	**%**	**Age-adjusted OR (95% CI)^a^**
*Education*				
Illiterate or primary^b^	247	37	15.0	1
Junior high school	336	51	15.2	1.0 (0.6–1.7)
Senior high school and above	79	10	12.7	0.8 (0.3–1.7)
*p for trend*=*0.64*				
				
*Age at first sexual intercourse (years)*				
⩾21^b^	209	34	16.3	1
19–20	293	47	16.0	0.9 (0.6–1.5)
⩽18	160	17	10.6	0.5 (0.2–1.0)
*p for trend*=*0.08*				
				
*Lifetime number of sexual partners*				
1^b^	545	85	15.6	1
⩾2	117	13	11.1	0.7 (0.3–1.2)
				
*Husband's extramarital sexual relationships*				
No^b^	583	88	15.1	1
Yes	79	10	12.7	0.8 (0.4–1.7)
				
*Age at menarche (years)*				
⩽14^b^	235	36	15.3	1
15–16	225	32	14.2	0.7 (0.4–1.2)
⩾17	202	30	14.9	0.7 (0.4–1.2)
*p for trend*=*0.18*				
				
*Menopausal status^c^*				
Premenopause^b^	90	18	20.0	1
Menopause <50 years old	106	17	16.0	0.7 (0.3–1.7)
Menopause ⩾50 years old	72	8	11.1	0.5 (0.2–1.3)
				
*No. of births*				
0	15	1	6.7	0.7 (0.1–5.7)
1^b^	154	16	10.4	1
2	336	54	16.1	1.3 (0.6–2.7)
⩾3	157	27	17.2	1.5 (0.6–3.7)
*p for trend*=*0.31*				
				
*Intrauterine device use*				
Never^b^	308	44	14.3	1
Ever	354	54	15.3	1.1 (0.7–1.7)
				
*Tubal ligation*				
No^b^	284	33	11.6	1
Yes	378	65	17.2	1.4 (0.8–2.3)

a Adjusted for age (10-year groups);

b Reference category;

c Women ⩾45 years only (*n*=268). OR=odds ratio; CI=confidence interval.
